# ETHNOPRED: a novel machine learning method for accurate continental and sub-continental ancestry identification and population stratification correction

**DOI:** 10.1186/1471-2105-14-61

**Published:** 2013-02-22

**Authors:** Mohsen Hajiloo, Yadav Sapkota, John R Mackey, Paula Robson, Russell Greiner, Sambasivarao Damaraju

**Affiliations:** 1 Department of Computing Science, University of Alberta, Edmonton, Alberta, Canada; 2 Alberta Innovates Centre for Machine Learning, University of Alberta, Edmonton, Alberta, Canada; 3 Department of Laboratory Medicine and Pathology, University of Alberta, Edmonton, Alberta, Canada; 4 Department of Oncology, University of Alberta, Edmonton, Alberta, Canada; 5 Cancer Care, Alberta Health Services, Edmonton, Alberta, Canada; 6 Department of Agricultural, Food and Nutritional Sciences, University of Alberta, Edmonton, Alberta, Canada

## Abstract

**Background:**

Population stratification is a systematic difference in allele frequencies between subpopulations. This can lead to spurious association findings in the case–control genome wide association studies (GWASs) used to identify single nucleotide polymorphisms (SNPs) associated with disease-linked phenotypes. Methods such as self-declared ancestry, ancestry informative markers, genomic control, structured association, and principal component analysis are used to assess and correct population stratification but each has limitations. We provide an alternative technique to address population stratification.

**Results:**

We propose a novel machine learning method, ETHNOPRED, which uses the genotype and ethnicity data from the HapMap project to learn ensembles of disjoint decision trees, capable of accurately predicting an individual’s continental and sub-continental ancestry. To predict an individual’s continental ancestry, ETHNOPRED produced an ensemble of 3 decision trees involving a total of 10 SNPs, with 10-fold cross validation accuracy of 100% using HapMap II dataset. We extended this model to involve 29 disjoint decision trees over 149 SNPs, and showed that this ensemble has an accuracy of ≥ 99.9%, even if some of those 149 SNP values were missing. On an independent dataset, predominantly of Caucasian origin, our continental classifier showed 96.8% accuracy and improved genomic control’s λ from 1.22 to 1.11. We next used the HapMap III dataset to learn classifiers to distinguish European subpopulations (North-Western vs. Southern), East Asian subpopulations (Chinese vs. Japanese), African subpopulations (Eastern vs. Western), North American subpopulations (European vs. Chinese vs. African vs. Mexican vs. Indian), and Kenyan subpopulations (Luhya vs. Maasai). In these cases, ETHNOPRED produced ensembles of 3, 39, 21, 11, and 25 disjoint decision trees, respectively involving 31, 502, 526, 242 and 271 SNPs, with 10-fold cross validation accuracy of 86.5% ± 2.4%, 95.6% ± 3.9%, 95.6% ± 2.1%, 98.3% ± 2.0%, and 95.9% ± 1.5%. However, ETHNOPRED was unable to produce a classifier that can accurately distinguish Chinese in Beijing vs. Chinese in Denver.

**Conclusions:**

ETHNOPRED is a novel technique for producing classifiers that can identify an individual’s continental and sub-continental heritage, based on a small number of SNPs. We show that its learned classifiers are simple, cost-efficient, accurate, transparent, flexible, fast, applicable to large scale GWASs, and robust to missing values.

## Background

### Single nucleotide polymorphisms

Single nucleotide polymorphisms (SNPs) as single base substitutions in DNA are the most common type of genetic variation in humans. SNPs are evolutionarily conserved and heritable. They give rise to one or more allelic variations at a loci and may confer phenotypic variance. Polymorphisms result from the evolutionary processes, and are modified by natural selection. They are common in nature and are related to biodiversity, genetic variation, and adaptation [1]. To date, millions of human SNPs have been identified and recorded in public databases such as dbSNP [2] or Ensembl [3].

### Genome wide association studies

A genome wide association study (GWAS) is an examination of a large set of common genetic variants, such as SNPs, over a set of “labeled” individuals, seeking variants that are associated with a phenotype, such as disease susceptibility, disease prognosis or drug response under the “Common Disease-Common Variant” hypothesis [4,5]. A GWAS normally compares the DNA of two groups of participants: subjects who expressed a phenotype (cases) versus subjects who did not (controls). Here, the researcher compares the values of each individual feature (e.g., specific SNP) in the cases, with the corresponding values for this feature in the controls. If the range of values in these subgroups is significantly different, this feature is said to be associated with the phenotype. In contrast to *candidate* gene polymorphism studies which test only a few pre-defined genetic regions, GWASs investigate the entire genome [6,7]. The database of genotypes and phenotypes (dbGaP) [8] and the catalogue of published GWASs [9] archive and distribute the findings from GWASs to the broader scientific community.

### Population stratification

Population stratification (aka population structure) is the presence of a systematic difference in allele frequencies between populations or subpopulations possibly due to different ancestry. We observe population stratification because of the differences in social history, ancestral patterns of geographical migration, mating practices, reproductive expansions and bottlenecks of different human subpopulations [10].

### Population stratification in GWASs

While conducting a GWAS, a major concern is the possibility of inducing false positive or false negative associations between a SNP and the phenotype due to population stratification. This has motivated many researchers to consider techniques to address population stratification problem. As a pre-processing step in GWAS, these techniques either exclude some of the study subjects to alleviate the problem or adjust some of the SNPs to correct for population structure [11]. Here we review some of the standard techniques used to deal with population stratification problem in GWASs and discuss their limitations:

#### Self-declared ancestry

Many studies ask subjects to identify their own ethnicity, by reporting their ancestry and country of origin. Then they address the problem of population stratification by including the cases and controls that have the same self-reported ancestry and by excluding other subjects from the GWAS. However this method is sometimes misleading as some people might not know their full lineage information, or simply be mistaken. Furthermore, self-declared ancestry is not always sufficient to control population stratification as nearly all populations are confounded by genetic admixture at some level [12].

#### Ancestry informative markers

Some projects attempt to estimate ancestry using a panel of ancestry informative markers (AIMs) that show the highest absolute value difference in allele frequency between two ancestral populations. A small set (typically tens to hundreds) of well-established AIMs can perfectly distinguish continental differences between individuals [13-16]; however, panels of AIMs, described thus far, are less informative in detecting sub-continental differences in closely related populations such as Europeans [17-25].

#### Genomic control

A widely used approach to evaluate whether a dataset is confounded due to population stratification involves computing the genomic control λ, which is defined as the median χ^2^ (1 degree of freedom) association statistic across SNPs, divided by its theoretical median under the null distribution. A value of λ ≈ 1 indicates no stratification, whereas λ > 1 indicates population stratification or other confounders [26-29]. Despite its widespread application, genomic control method has a fundamental limitation. In the real world, some markers differ in their allele frequencies across ancestral populations more than others while the genomic control corrects for stratification by adjusting association statistics at each marker by a uniform overall inflation factor. This uniform adjustment is not sufficient to deal with both markers that have strong differentiation across ancestral populations and also those with smaller differentiation.

#### Structured association

Structured association techniques are unsupervised learning (clustering) methods such as STRUCTURE [30] which is based on a Bayesian framework and latent class analysis [31] which is based on maximum-likelihood that assign subjects of a case–control study cohort to discrete subpopulations based on their inter-cluster similarities and intra-cluster dissimilarities [32,33]. Although structured association methods have the advantage of assigning samples into meaningful population groups, they cannot be applied to GWAS datasets because of their intensive computational cost on large datasets provided by recent high-throughput measurements.

#### Principal component analysis

Techniques based on principal component analysis (PCA) [34-36], like EIGENSTRAT [34], are currently the state-of-the-art methods used in GWASs for population stratification correction. The EIGENSTRAT algorithm applies PCA to genotype data to infer continuous axes of genetic variations represented by principal component vectors and then adjusts genotypes and phenotype by amounts attributable to ancestry along each axis. Despite the widespread application of such PCA-based techniques, they have some disadvantages: First, they are not cost-efficient since they require genotyping thousands to millions of markers to be able to calculate principal component vectors. Second, to infer ancestry of subjects they apply PCA, a black-box model, which is not human readable (transparent). Third, as high-throughput measurements produce many missing values, the straightforward PCA does not apply, leading EIGENSTRAT to use missing value imputation. However, such imputation techniques are problematic in population genetics as they ignore inter-individual and inter-ethnic variations, meaning such imputed datasets can lead to spurious association findings [37]. Fourth, the genotyping errors (GEs) that arise in high-throughput SNP measurements are a major issue in association studies [38-44] and substantially affect the efficiency of PCA-based methods like EIGENSTRAT [45].

### The purpose of our research study

In this paper, we introduce a novel method, EHNOPRED, for producing models that can accurately place subjects within continental and sub-continental populations, by applying a supervised learning (classification) technique to datasets from the second and third phases of the international HapMap project [46]. The resulting classifiers can help correct population stratification in association studies, overcoming some of the limitations of the conventional methods listed above. First, self-declared ancestry information is problematic, except possibly for isolated populations with extensive inbreeding. ETHNOPRED does not rely on self-declared ancestry information and analyzes an individual’s genome to properly identify his/her ancestry. Second, while small panels of AIMs for continental population identification are designed, panels of AIMs for sub-continental population identification, if designable, either are less informative or use a large set of markers. However, ETHNOPRED produces accurate classifiers not only for continental population detection but also for sub-continental population detection using a small number of markers. Third, ETHNOPRED is not relying on the assumption of the genomic control method that all markers contribute equally to population stratification and instead benefits from the fact that different markers contribute to population differences in different degrees. Fourth, unlike structured association methods, ETHNOPRED classifiers are fast and easily applicable to the large GWAS datasets generated by high-throughput measurement techniques like microarrays and next generation sequencers. Fifth, ETHNOPRED classifiers require genotyping of only tens to hundreds of SNPs for accurate population identification. Hence they are simpler and more cost-efficient in comparison to PCA-based methods, which require genotyping of thousands to millions of SNPs. Sixth, PCA-based methods like EIGENSTRAT are substantially affected by the genotyping errors arisen in high-throughput SNPs measurements. However, low-throughput SNP measurements of tens to hundreds of SNPs required by ETHNOPRED classifiers may be easily validated on independent genotyping platforms to rule out genotyping errors and assess concordance of genotype calls across independent platforms. Once these criteria are established, these selected SNP panels could be used to identify population stratification across projects sharing similar cases and control cohorts in molecular epidemiological studies. Seventh, ETHNOPRED classifiers are a set of easy-to-read rules. Thus unlike PCA-based methods, these classifiers are transparent, and so can provide insight into the population classification problem they are dealing with. Eighth, unlike PCA-based methods, ETHNOPRED classifiers do not require any kind of imputation to handle missing values. ETHNOPRED classifiers are robust to missing values as their ensemble structure allows them the flexibility to deal with missing SNPs by simply removing some decision trees, and still remain able to accurately identify ancestry.

## Methods

### Datasets

Our objective is to build predictive tools to determine an individual’s continental and sub-continental ancestry based on the values of a small set of his/her SNPs. We develop this tool by applying supervised learners to datasets from the second and third phases of the international HapMap project. The HapMap project is a multi-country effort to identify and catalogue genetic similarities and differences in human beings and to determine the common patterns of DNA sequence variations in the human genome. It is developing a map of these patterns across the genome by determining the genotypes of more than a million sequence variants, their frequencies and the degree of association between them, in DNA samples from subpopulations with ancestry from East and West Africa, East Asia, North and West Europe, and North America.

The HapMap phase II datasets, released in 2007, contained 270 subjects – including 90 Utah residents with ancestry from Northern and Western Europe (CEU), 90 Yorubans from Ibadan, Nigeria (YRI), and a mixture of 45 Japanese in Tokyo and 45 Han Chinese in Beijing (JPT/CHB) – each genotyped on an Affymetrix SNP array 6.0 platform, measuring 906600 SNPs. We utilize the HapMap II datasets to build a predictive model for inferring the continental ancestry origins (West Africa vs. East Asia vs. North-West Europe) of an individual. We apply the resulting classifier to a dataset of 696 breast cancer study subjects (348 breast cancer cases and 348 apparently healthy controls) from Alberta, Canada, genotyped on the same Affymetrix SNP array platform. We have self-declared ancestry of these 348 control individuals. These study subjects provided written informed consent and the study was approved by the Alberta Cancer Research Ethics Committee of the Alberta Health Services [47].

**Figure 1:**
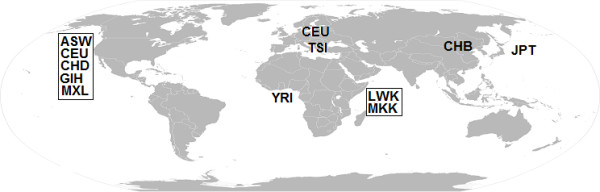
**Geographic map of the HapMap phase III world populations.** ASW = Southwest USA residents with African ancestry; CEU = Utah residents with Northern and Western European ancestry; CHB = Han Chinese in Beijing, China; CHD = Chinese in Metropolitan Denver, Colorado; GIH = Gujarati Indians in Houston, Texas; JPT = Japanese in Tokyo, Japan; LWK = Luhya in Webuye, Kenya; MKK = Maasai in Kinyawa, Kenya; MXL = Mexicans in Los Angeles, California; TSI = Toscani in Italia; YRI = Yoruba in Ibadan, Nigeria.

The HapMap phase III datasets, released in 2009, contained 1458387 SNPs of 1397 subjects including 87 Southwest USA residents with African ancestry (ASW), 165 Utah residents with ancestry from Northern and Western Europe (CEU), 137 Han Chinese in Beijing, China (CHB), 109 metropolitan Denver, Colorado residents with Chinese ancestry (CHD), 101 Gujarati Indians in Houston, Texas (GIH), 113 Japanese in Tokyo, Japan (JPT), 110 individuals from Luhya tribe in Webuye, Kenya (LWK), 86 Los Angeles, California residents with Mexican ancestry (MXL), 184 individuals from Maasai tribe in Kinyawa, Kenya (MKK), 102 Toscani Italians (TSI), and 203 Yorubans in Ibadan, Nigeria (YRI). Figure 1 shows the geographic map of the HapMap III world populations. We utilize the HapMap III datasets to build predictive models for infering sub-continental ancestry origins of Africans (LWK vs. MKK vs. YRI), Europeans (CEU vs. TSI), East Asians (CHB vs. JPT), North Americans (ASW vs. CEU vs. CHD vs. GIH vs. MXL), Kenyans (LWK vs. MKK), and Chinese (CHB vs. CHD).

### Pre-processing

The allele with the dominant occurrence within a population is called the major allele (A), while the allele occurring less frequently is called the minor allele (B). Together, the alleles from paternal and maternal chromosomal loci can produce three distinct genotypes: When both alleles (*ie*, inherited from both parents) are the major alleles (A_A), the genotype is called wild type homozygous; when both the inherited alleles are minor (B_B), the genotype is called variant type homozygous; and when the two alleles are different (A_B), the genotype is called heterozygous.

To build our continental population classifier, we first identified the relevant SNPs from the HapMap II dataset, by removing a SNP if (a) it has a NoCall for *any* of the 270 subjects; (b) it is located on the X, Y, mitochondria (MT), or on an unknown chromosome; or (c) its genotype frequency deviates significantly from Hardy-Weinberg equilibrium (HWE) proportions, tested with Pearson’s chi-squared (χ^2^) test (nominal p-value < 0.05) [48]. We used criteria (a) to train our model using SNPs without missing values; (b) so the tool would be applicable to anyone, regardless of gender; and (c) by reasoning that observed genotype frequencies that deviate from HWE do not match the expected distributions of alleles, and hence are not reliable. These pre-processing steps removed a total of 295454 SNPs, leaving 611146 SNPs amenable for further scrutiny. Table 1 summarizes the statistics of the SNPs removed in the pre-processing steps, applied on HapMap II datasets.

**Table 1:** Pre-processing statistics of for continental population classification problem based on HapMap Phase II samples

**SNP groups**	**Number of SNPs**
All SNPs	906600
SNP with Call Rate < 100%	186578
SNPs on Non-autosomal Chromosomes	38306
SNPs Deviated from HWE	184854
Filtered SNPs	295454
Unfiltered SNPs	611146

To build our sub-continental population classifiers, we followed similar filtering criteria on HapMap III dataset. These pre-processing steps respectively removed 841790, 565554, 575492, 931993, 677326, and 629023 SNPs, and left 616597, 892833, 882895, 526394, 781061, and 829364 SNPs amenable for further analysis in African population classification problem, East Asian population classification problem, European population classification problem, North American population classification problem, Kenyan population classification problem, and Chinese population classification problem. Table 2 summarizes the statistics of the SNPs removed in the pre-processing steps, applied on HapMap III datasets.

**Table 2:** Pre-processing statistics of HapMap phase III datasets and sub-continental population classification problems

**Dataset/Problem**	**Samples**	**All SNPs**	**SNPs with call rate < 100%**	**SNPs on Non-autosomal Chr.**	**SNPs deviated from HWE**	**Filtered SNPs**	**Unfiltered SNPs**
**ASW**	87	1458387	214898	34554	94234	298524	1159863
**CEU**	165	1458387	376531	34554	81633	427638	1030749
**CHB**	137	1458387	353208	34554	77028	423270	1035117
**CHD**	109	1458387	352031	34554	77111	421328	1037059
**GIH**	101	1458387	234863	34554	85463	314376	1144011
**JPT**	113	1458387	271105	34554	75502	337033	1121354
**LWK**	110	1458387	365638	34554	97174	425375	1033012
**MKK**	184	1458387	411395	34554	105490	471384	987003
**MXL**	86	1458387	311704	34554	86910	387207	1071180
**TSI**	102	1458387	268916	34554	81919	326585	1131802
**YRI**	203	1458387	423100	34554	94449	476513	981874
**European**	267	1458387	493449	34554	137488	575492	882895
**East Asian**	250	1458387	475217	34554	129695	565554	892833
**African**	497	1458387	742671	34554	228268	841790	616597
**North American**	548	1458387	803678	34554	306572	931993	526394
**Kenyan**	294	1458387	590202	34554	170547	677326	781061
**Chinese**	246	1458387	538224	34554	131394	629023	829364

### Predictive modelling

Machine learning provides a variety of statistical, probabilistic, and optimization techniques to analyze and interpret data, which allow computers to autonomously learn from past examples by finding patterns to form predictive models – often finding hard-to-discern patterns, from noisy and complex datasets [49-51]. Machine learning has been applied successfully in many areas: Baldi and Brunak [52], Larranga et al. [53], and Tarca et al. [54] each surveyed various applications of machine learning in biology, medicine, and genetics including gene finding [55], eukaryote promoter recognition [56], protein structure prediction [57], pattern recognition in microarrays [58], gene regulatory response prediction [59], and protein/gene identification in text [60]. Herein, we learn a sequence of CART decision trees for continental and sub-continental population identification [61,62]. While machine learning provides many systems for learning classifiers, we focus on decision trees as these learners are easy to use (as they do not require the user to provide any input parameters) and relatively fast to train, and the resulting classifiers run quickly and are easy to interpret (which may explain why they are widely applied in biological/medical domains).

“Ensemble learning” refers to a class of machine learning methods that combine the individual decisions of a set of learned “base predictors” to obtain a better predictive performance [63]. In general, an ensemble of predictors will be more accurate than any of its individual members if the constituent predictors are individually accurate and collectively diverse [64]. Ensemble models have been successfully applied on high-dimensional datasets generated by novel “omics” measurements, such as gene expression microarrays [65,66]. Many ensemble techniques – such as bagging, boosting, AdaBoost, and stacking – rely on manipulation of the input dataset by sampling of subjects or sampling of features, then learning individual base classifiers on these subsets of the input dataset [67]. While the main goal of ensemble predictors is to produce an *accurate* classifier (as the ensemble can sometimes overcome the over-fitting problem reported for decision trees in high-dimensional problems [68]), we used this approach to produce a classifier that is *robust* to missing SNP values. Our system therefore learns a set of *disjoint* trees; we later explain how this allows the classifier to predict the label of a subject, even if that subject is missing many SNP values.

**Figure 2:**
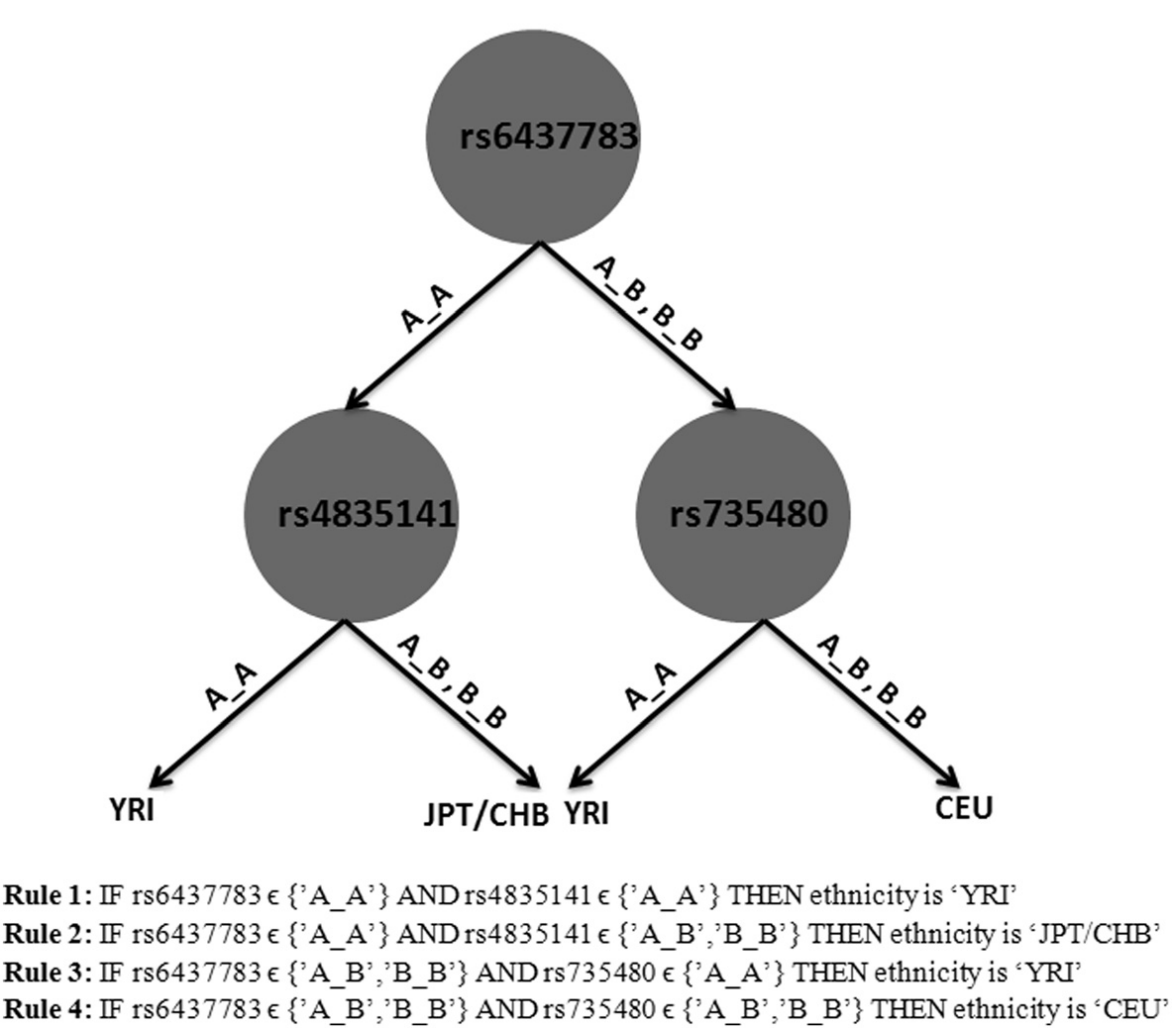
**The first decision tree and associated rule-set of the continental classifier produced by ETHNOPRED algorithm.** The decision tree uses 3 internal nodes (SNPs) acting as decision criterions and 4 external nodes (populations) demonstrating decisions. The number of rules in the relevant rule-base is equal to the number of external nodes of the decision tree.

Here we explain how ETHNOPRED learns an ensemble of disjoint decision trees, focusing on continental population classifier case. It first applies the CART learning algorithm to the dataset of 270 subjects over the 611146 SNPs mentioned above, to produce the decision tree (Figure 2) with 3 internal nodes (each a condition on a specific SNP) and 4 leaf nodes (class labels), corresponding to the 4 rules shown in Figure 2. It then removes these 3 SNPs from the list of 611146 SNPs and applies the same CART decision tree learning algorithm to the dataset of 270 subjects and the remaining 611143 SNPs, to produce a second decision tree. We repeat this algorithm, each time removing the SNPs used in the previous trees, to produce the next decision tree.

The ETHNOPRED continental population classifier learns N = 29 disjoint decision trees. We explain below that N = 29 guarantees that this system is robust against missing SNP values – that is, based on some simple assumptions, we anticipate that at least 99.9% of the subjects will include calls on the SNPs needed to “match” several decision trees; enough trees that the resulting sub-ensemble will be at least 99.9% accurate. This analysis appears below.

**Figure 3:**
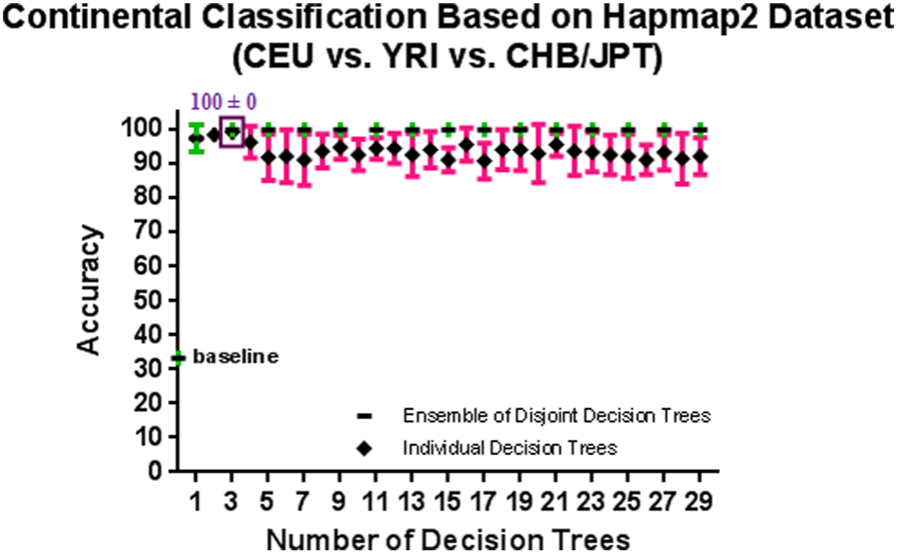
**A comparison of 10-fold cross validation accuracy of individual decision trees and ensembles of disjoint decision trees of variable size in continental population classification problem using HapMap phase II datasets.** An ensemble of 3 disjoint decision trees involving 10 SNPs has a 10-fold cross validation accuracy of 100% which is significantly better than the baseline accuracy of 33.3%.

Additional file 1: Appendix A and Figure 3 show the estimated accuracies of the first k decision tree: the first tree, alone, is 97.41% and the ensemble classifier using the first 3 decision trees is 100%. If accuracy was our only concern, our ensemble classifier would just use these 3 decision trees, involving its 10 SNPs. However, this 3 decision tree system can only classify a subject if that subject includes values for (essentially) all 10 SNPs. Missing genotype data is a common problem in genotyping experiments, due to assay design failures, platform specific differences in the SNPs analyzed or due to hybridization artifacts in these high-throughput array platforms [69]. Here, we show that N = 29 decision trees are sufficient, under mild assumptions, to obtain an accuracy (Acc) of ≥ 99.9% with 99.9% confidence (C), even considering missing SNPs: We trained 30 disjoint decision trees and found the average number of SNPs used in these 30 decision trees is n = 154/30 ≈ 5.13. We then assumed that, for the Affymetrix genome wide SNP array 6.0 platform, NoCall’s are independent from one SNP to another, and that the probability that a SNP value will be a NoCall is at worst u = 0.1 (based on assessment on the HapMap II dataset). This means that the probability that a subject will include all of the SNPs for a decision tree is p ≤ (1-u)^n^ = 0.9^5.13^ = 0.59049, and so the probability that a subject will not include all of the SNPs of a decision tree is at least q = 1 – p = 0.40951. We now ask how many decision trees (m) are needed to insure that the average accuracy (Acc) of any subset of m trees is at least 99.9%. We therefore considered a sampling of ensembles of size 1 (i.e., individual decision trees) and calculated the average 10-fold cross validation accuracy. We next computed the average 10-fold cross validation accuracy over a sample of pairs of decision trees; then over triples, and so forth, for i = 1..30 (Table 3). We found that m = 9 is sufficient to obtain an average 10-fold cross validation accuracy (Acc) of 99.9%.

**Table 3:** The average 10-fold cross validation accuracy of ensembles of size m, for m = 1..30 in continental population classification problem

**Number of models in the ensemble (m)**	**Number of ensembles**	**The average 10-fold cross validation accuracy of ensembles of size m (Acc)**
1	30	95.38
2	435	91.34
3	4060	98.36
4	27405	97.03
5	142506	99.32
6	593775	98.81
7	2035800	99.67
8	5852926	99.44
9	14300000	99.93
10	30000000	99.92
11	54600000	99.98
12	86500000	99.96
13	120000000	99.99
14	145000000	99.99
15	155000000	99.99
16	145000000	99.99
17	120000000	99.99
18	86500000	99.99
19	54600000	99.99
20	30000000	99.99
21	14300000	99.99
22	5852926	99.99
23	2035800	99.99
24	593775	99.99
25	142506	99.99
26	27405	99.99
27	4060	99.99
28	435	99.99
29	30	99.99
30	1	100

The next challenge was in determining how many trees (N) are necessary, to be confident that the SNPs for 99.9% of all subjects will include calls on all of the SNPs for at least 9 trees.The probability of having at least m decision trees with no missing SNPs, given N decision trees, with probability p that a decision tree includes only specified SNPs, is:

(1) $$C=(1-\overset{8}{\underset{i=1}{\sum }}\left(\begin{array}{c} N\\ i \end{array}\right)*p^{i}*{\left(1-p\right)}^{N-i})$$

Table 4 shows the values for C based on different values for N; here, we see N = 29 decision trees is sufficient to have 99.9% confidence (C) that a subject will include all of the SNPs in at least m = 9 decision trees, which our earlier experiments show is sufficient to produce an accuracy of ≥ 99.9%. Additional file 2: Appendix B summarizes this analysis.

**Table 4:** The confidence of having m = 9 decision trees without missing SNPs for N = 1..30 in continental population classification problem

**Number of decision trees (N)**	**Confidence of having m = 9 decision trees with no missing SNPs (C)**
1	0
2	0
3	0
4	0
5	0
6	0
7	0
8	0
9	0.873
10	4.09
11	10.676
12	20.566
13	32.716
14	45.652
15	58.013
16	68.86
17	77.744
18	84.616
19	89.682
20	93.265
21	95.71
22	97.328
23	98.369
24	99.023
25	99.424
26	99.666
27	99.809
28	99.892
29	99.94
30	99.967

### Models’ usage for population stratification correction

For each continental and sub-continental ancestry identification problem, the pre-processing and predictive modeling steps produce a model (i.e., in the case of continental classification problem, the model is an ensemble of 29 decision trees) that can be used to classify novel subjects. For example, in continental population identification, we need to only find the values {A_A, A_B, B_B, NoCall} of the relevant 149 SNPs, then hand this set of 149 values to each of the 29 decision trees. Each tree involves a small number of SNPs (typically 3–7); if they are all specified (that is, none are “NoCall”) for a novel subject, this tree will produce a predicted label – one of the three ethnicity groups: CEU, YRI, or CHB/JPT. If not, the tree makes no prediction. This will lead to a set of at-most-29 predicted ethnicity values for this subject. As no human population is homogenous, given a novel subject with unknown ancestry, our model can provide a vector of population inclusion probabilities.

For example, when classifying a novel person with the initial continental classification, imagine 15 trees vote for CEU, 4 for YRI, 8 for JPT/CHB, and 2 are silent; this would produce the vector (15/27, 4/27, 8/27). These vector-valued predictions provide flexibility for researchers conducting a GWAS, as they can then, for example, define cut-off criterion for including a subject within a population under study. For each subject, continental classifier then returns, as ethnicity label, the ethnicity with the largest number of trees. In the *Results* section, we explain such panels for resolving the population stratification problem in closely related populations within a continent or a country as well.

### Evaluation

We built the ETHNOPRED classifiers using HapMap II and HapMap III datasets as training data. Before using each classifier, we estimated its quality using a 10-fold cross validation (CV) [70]. This meant partitioning the training dataset into 10 disjoint folds. Each time we used nine of these folds (9/10^th^ of data) as training set for learning a sequence of decision trees, applying the algorithm explained in the *Predictive Modeling* section. We then used the remaining fold (1/10^th^ of data) as a test set; here to compute, for each subject, class labels (one from each decision tree), and also the majority vote over these model (corresponding to the ensemble classifier). As we knew the true label for these subjects, we then obtained an accuracy score (the percentage of correct predictions over the total number of predictions) for each of the disjoint decision trees and for the final ensemble. We repeated this process 10 times, each time measuring accuracy of the predictors on a different fold. We estimated the final accuracy of the decision trees and ensemble model as an average of these 10 folds, with variance based on the spread of these 10 numbers. We used a similar way to evaluate the quality of the ETHNOPRED(k) classifier, where each such classifier was involved in returning the majority vote over subsequence of k individual decision trees.

## Results and discussion

### Continental ancestry identification

Table 1 summarizes the statistics of the SNPs removed in the pre-processing step, which recall filtered out each SNP with a call rate of less than 100%, or that are located on X, Y, MT, or an unknown chromosome, or deviated from the HWE; this removed 295454 SNPs, leaving 611146 SNPs for further analyses.

The final ensemble model, learned from all 270 subjects of the HapMap Phase II datasets, was composed of 29 disjoint decision trees, which each involved between 3 to 7 SNPs and between 4 to 8 leaf nodes/rules. This corresponds to a total of 178 rules involving 149 SNPs in the ensemble model (see Additional file 3: Appendices C, Additional file 4: Appendix D and Additional file 5: Appendix E). Additional file 1: Appendix A and Figure 3 present the 10-fold cross validation (CV) accuracy of the disjoint decision trees built based on the ETHNOPRED algorithm showing the mean of the 10-fold CV accuracy of these models was between 90.7% and 99.3%. We see that the ensemble over only the first tree had a mean accuracy of 97.4%; the accuracy decreased (albeit insignificantly) to 95.9% by adding the second tree; the ensemble over 3 (or more) trees was 100% accurate. While adding additional trees to the ensemble did not improve the accuracy, our approach did increase its robustness to missing SNP values, as it means ETHNOPRED can produce a classification label even if the subject did not have calls on all 149 SNPs. Recall that ETHNOPRED can classify most subjects with missing SNP values as it can ignore any tree that includes missing SNPs, and returns as label the majority vote of the remaining trees.

To further assess the accuracy of ETHNOPRED, we also used a hold-out set of 696 breast cancer subjects (348 breast cancer cases and 348 controls) genotyped in Alberta, Canada. We had self-declared ethnicity labels for the control subjects. Here, we compared our ETHNOPRED against the commonly-used EIGENSTRAT system, in terms of the prediction accuracy and genomic control inflation factor (λ) improvement. Here, we extracted the values of ETHNOPRED’s 149 SNPs for each subject. Note that 17 of these 149 SNPs had NoCalls for at least one subject. For each subject, each of ETHNOPRED’s 29 decision trees predicted the subject’s ethnicity to be one of “CEU”, “YRI”, “JPT/CHB”, or “Missing”. Continental classifier then calculates the covariate probability vector and returns the ethnicity with the majority vote as the predicted label for that subject. Additional file 6: Appendix F summarizes ETHNOPRED output for test dataset of 696 subjects. Prior knowledge of the subjects’ ethnicity labels, when available, would help assess the predictive accuracies of ETHNOPRED (or EIGENSTRAT) – eg, many previously published studies (including our [45]) have used the HapMap subjects’ self-declared ethnicity label to evaluate their ethnicity classifiers. We extrapolated this logic to calculate the prediction accuracies of ETHNOPRED over 348 control subjects, based on their self-declared ethnicity. Additional file 7: Appendix G summarizes the subjects’ ethnicity labels, classified by ETHNOPRED (and the number of decision trees involved), EIGENSTRAT, and self-declared ethnicity label. Table 5 shows that ETHNOPRED’s ethnicity classification matched closely with the subject’s self-reported ethnicity (96.8%); Table 6 provides similar statistics for EIGENSTRAT (97.4%). The ETHNOPRED classifier labels 677 subjects as “CEU”; we could therefore use only these subjects and exclude the other 19 subjects for which either “YRI” or “CHB/JPT” is the majority ancestry covariate. Then we computed the inflation factor using the Genomic Control method for these subjects. For the entire sample size of 696 unclassified subjects in the association study, the computed inflation factor was 1.22, whereas the inflation factor computed for the 677 subjects classified as “CEU” by ETHNOPRED was 1.11, and the inflation factor for the 623 subjects classified as “CEU” by EIGENSTRAT was 1.10. While ETHNOPRED’s learned classifier gives roughly the same improvement to the inflation factor as EIGENSTRAT, it offered the advantage of using a set of only 149 SNPs to achieve the classification of ethnicity label (CEU), which is significantly smaller than the 906,600 SNPs used by EIGENSTRAT.

**Table 5:** **Comparison of self-declared lineage information and ETHNOPRED’s result on 348 controls selected for a breast cancer susceptibility study of Caucasian women of Alberta, Canada**[45]

	**ETHNOPRED predicts as CEU**	**ETHNOPRED predicts as non-CEU**
**Self-declared lineage information as CEU**	330	0
**Self-declared lineage information as non-CEU**	11	7

**Table 6:** **Comparison of self-declared lineage information and EIGENSTRAT’s result on 348 controls selected for a breast cancer susceptibility study of Caucasian women of Alberta, Canada**[45]

	**EIGENSTRAT predicts as CEU**	**EIGENSTRAT predicts as non-CEU**
**Self-declared lineage information as CEU**	321	0
**Self-declared lineage information as non-CEU**	9	18

### Sub-continental ancestry identification

Table 2 summarizes the statistics of the SNPs filtered in the pre-processing step: those SNPs with a call rate of less than 100%, or located on X, Y, MT, or on an unknown chromosome, or deviated from the HWE; starting with 1458387 SNPs in the HapMap III dataset, this filtering removed 493449, 475217, 742671, 803678, 590202, and 538224 SNPs respectively in European, East Asian, African, North American, Kenyan, and Chinese population classification problems, and left 882895, 892833, 616597, 526394, 781061, and 829364 SNPs for further analyses.

**Figure 4:**
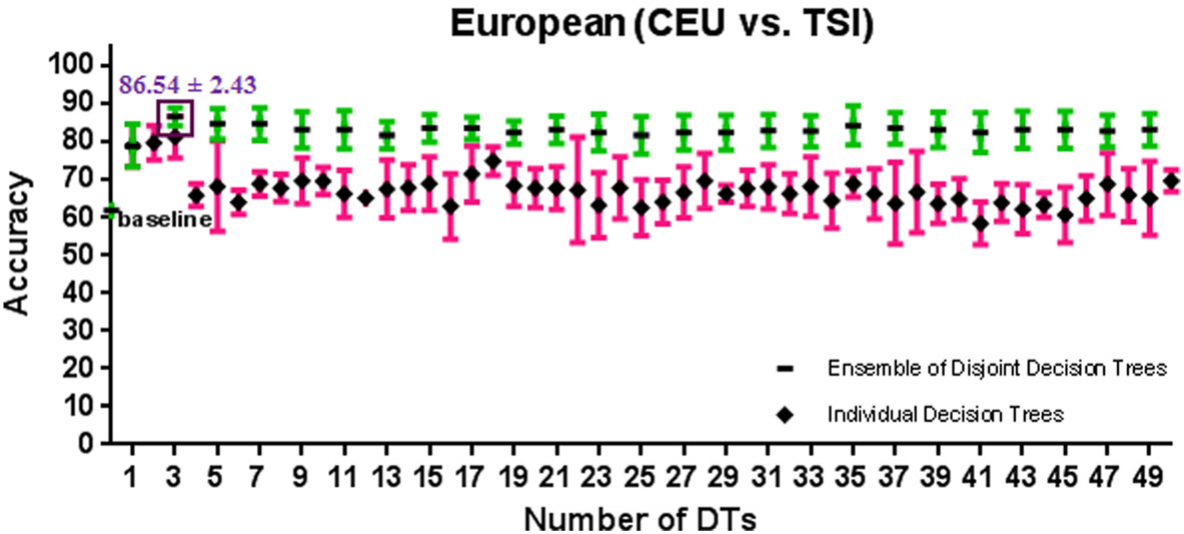
**A comparison of 10-fold cross validation accuracy of individual decision trees and ensembles of disjoint decision trees of variable size in European population classification problem using HapMap phase III datasets.** An ensemble of 3 disjoint decision trees involving 31 SNPs has a 10-fold cross validation accuracy of 86.5% ± 2.4% which is significantly better than the baseline accuracy of 61.8%.

**Figure 5:**
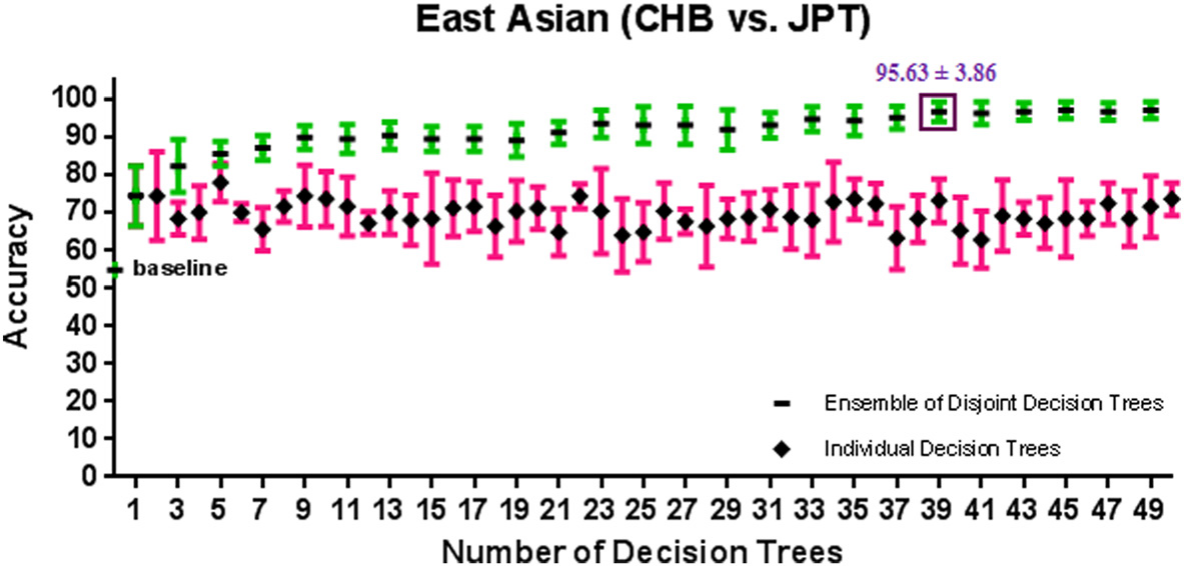
**A comparison of 10-fold cross validation accuracy of individual decision trees and ensembles of disjoint decision trees of variable size in East Asian population classification problem using HapMap phase III datasets.** An ensemble of 39 disjoint decision trees involving 502 SNPs has a 10-fold cross validation accuracy of 95.6% ± 3.9% which is significantly better than the baseline accuracy of 54.8%.

**Figure 6:**
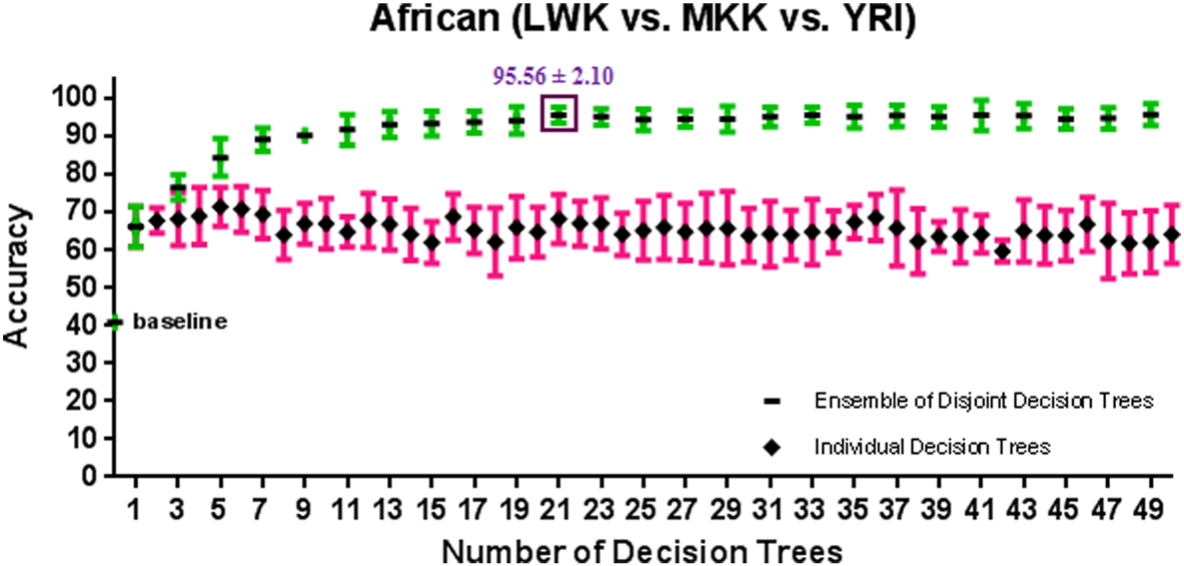
**A comparison of 10-fold cross validation accuracy of individual decision trees and ensembles of disjoint decision trees of variable size in African population classification problem using HapMap phase III datasets.** An ensemble of 21 disjoint decision trees involving 526 SNPs has a 10-fold cross validation accuracy of 95.6% ± 2.1% which is significantly better than the baseline accuracy of 40.8%.

**Figure 7:**
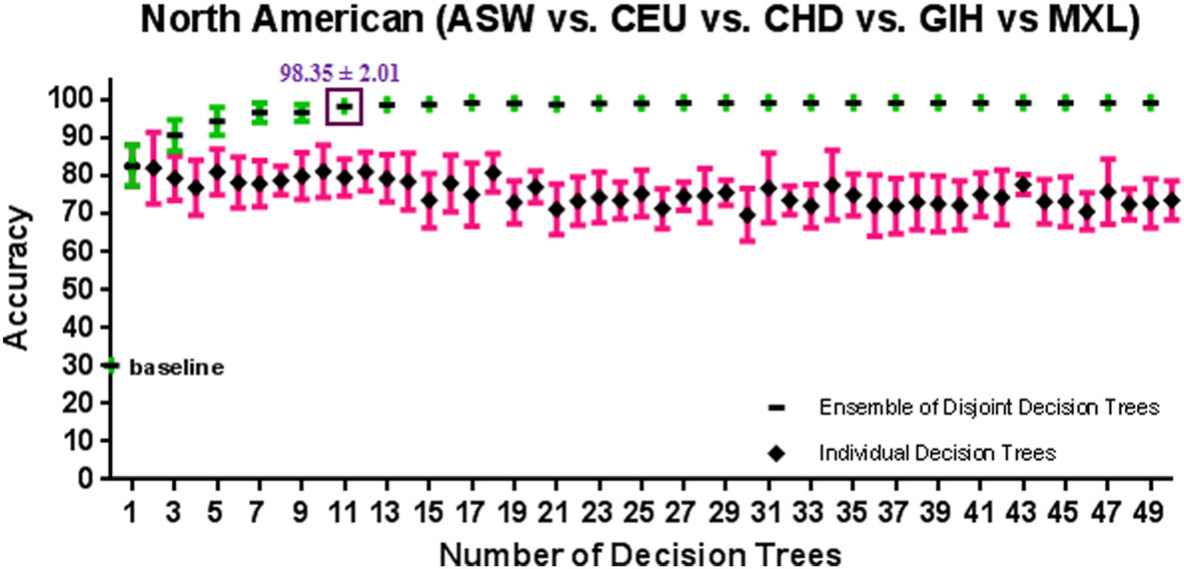
**A comparison of 10-fold cross validation accuracy of individual decision trees and ensembles of disjoint decision trees of variable size in North American population classification problem using HapMap phase III datasets.** An ensemble of 11 disjoint decision trees involving 242 SNPs has a 10-fold cross validation accuracy of 98.3% ± 2.0% which is significantly better than the baseline accuracy of 30.1%.

**Figure 8:**
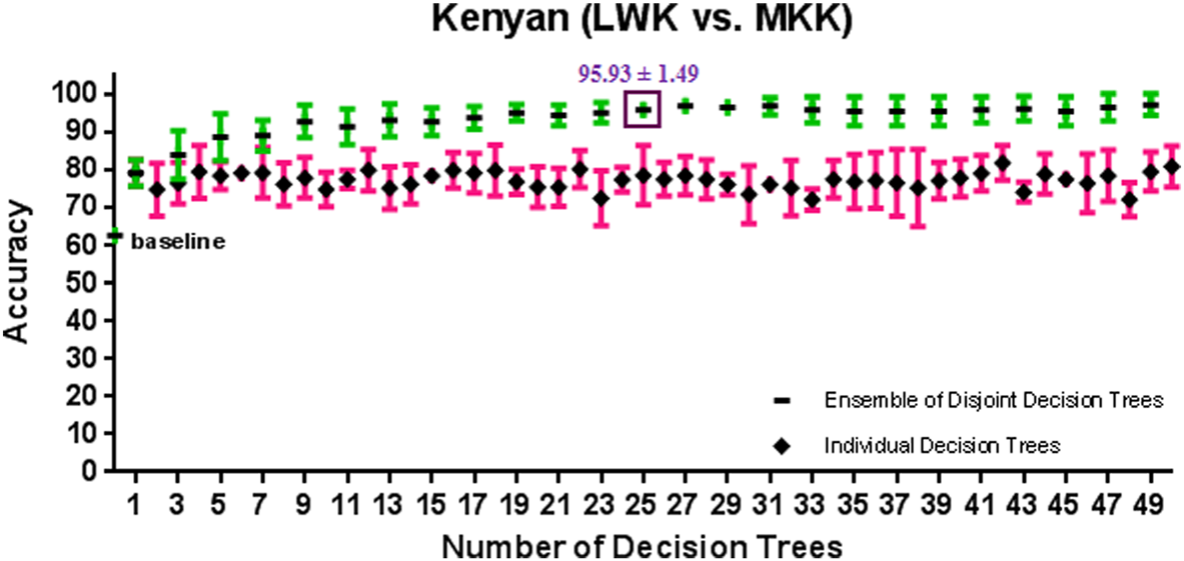
**A comparison of 10-fold cross validation accuracy of individual decision trees and ensembles of disjoint decision trees of variable size in Kenyan population classification problem using HapMap phase III datasets.** An ensemble of 25 disjoint decision trees involving 271 SNPs has a 10-fold cross validation accuracy of 95.9% ± 1.5% which is significantly better than the baseline accuracy of 62.6%.

**Figure 9:**
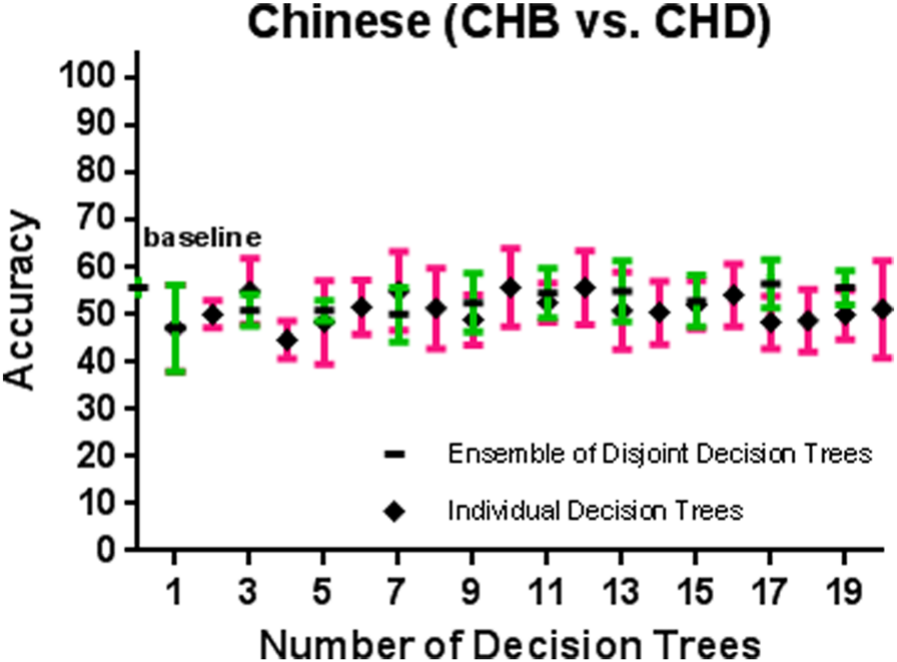
**A comparison of 10-fold cross validation accuracy of individual decision trees and ensembles of disjoint decision trees of variable size in Chinese population classification problem using HapMap phase III datasets.** We considered several individual decision trees and ensembles of various sizes, but none had 10-fold cross validation accuracy better than the baseline accuracy of 55.7%.

Table 7 summarizes the results of our study on these sub-continental population classification problems respectively for the case of European, East Asian, African, North American, Kenyan, and Chinese population classification problems. Additional file 1: Appendix A and Figures 4, 5, 6, 7, 8, and 9 show the 10-fold CV accuracy of the individual disjoint decision trees and ensembles of varying size built over those trees using the ETHNOPRED algorithm. The baseline accuracy calculated by simply classifying every subject to the majority class in each of these sub-continental identification problems is as follows: 61.8%, 54.8%, 40.8%, 30.1%, 62.6%, and 55.7%. In each of these problems, the accuracy of a single decision tree, using 10, 12, 23, 19, 11, and 15 SNPs, is as follows: 79.0% ± 5.6%, 74.4% ± 7.9%, 66.2% ± 5.3%, 82.7% ± 5.4%, 79.2% ± 3.5%, and 47.2% ± 9.1%. These accuracies are significantly better than the baseline accuracy in every case except the Chinese one. Regardless of the Chinese case, ensembles of 3, 39, 21, 11, and 25 decision trees using 31, 502, 526, 242, and 271 SNPs have accuracy equal to 86.6% ± 2.4%, 95.6% ± 3.9%, 95.6% ± 2.1%, 98.4% ± 2.0%, and 95.9% ± 1.5% which are all statistically significantly better than the accuracy of the individual decision trees in other sub-continental classification problems. While adding additional trees to these ensembles does not improve the accuracy, using the arguments described in *Predictive Modelling* section, these additional trees do increase its robustness to missing SNP values; our analysis shows that an ensemble of 15, 67, 157, 70, and 31 decision trees using 180, 877, 4236, 1643, and 341 SNPs guarantees both accuracy and robustness to missing values in these cases. Additional file 2: Appendix B summarizes this analysis and Additional file 4: Appendix D and Additional file 5: Appendix E show information related to the SNPs used for sub-continental population identification problems under the accuracy condition satisfaction and the robustness to missing values condition satisfaction paradigms. As mentioned above, ETHNOPRED is unable to produce a classifier that can distinguish between Chinese in Beijing and Chinese in Denver. We believe this is not a limitation of our algorithm given the fact that the first Chinese immigrant arrived in U.S. less than 200 years ago.

**Table 7:** Summary of the sub-continental classification problems results

**Sub-continental problem**	**Number of subjects, split**	**Number of SNPs**	**Baseline**	**DT1 (Number of SNPs), Accuracy**	**Minimal Number of DTs (Number of SNPs), Accuracy**	**Number of Robust DTs (Number of SNPs)**
European	267,	882895	61.8%	1 (10), 79.0% ± 5.6%	3 (31), 86.6% ± 2.4%	15 (180)
	CEU: 165					
	TSI: 102					
East Asian	250,	892833	54.8%	1 (12), 74.4% ± 7.9%	39 (502), 95.6% ± 3.9%	67 (877)
	CHB: 137					
	JPT: 113					
African	497,	616597	40.8%	1 (23), 66.2% ± 5.3%	21 (526), 95.6% ± 2.1%	157 (4236)
	LWK:110					
	MKK: 184					
	YRI: 203					
North American	548,	526394	30.1%	1 (19), 82.7% ± 5.4%	11 (242), 98.4% ± 2.0%	70 (1643)
	ASW: 87					
	CEU: 165					
	CHD: 109					
	GIH: 101					
	MXL: 86					
Kenyan	294,	781061	62.6%	1 (11), 79.2% ± 3.5%	25 (271), 95.9% ± 1.5%	31 (341)
	LWK: 110					
	MKK: 184					
Chinese	246,	829364	55.7%	1 (15), 47.2% ± 9.1%	- (−), ≤55.7%	- (−)
	CHB: 137					
	CHD: 109					

This table summarizes the result of our studies on various sub-continental classification problems. The “Number of Subjects, Split” column shows the total number of subjects, followed by the list of (ethnic-group; number) pairs, giving the name of each subgroups and its size here. The “Number of SNPs” column gives the number of SNPs used for this study. The “Baseline” column gives the baseline accuracy of just using the majority class. The “DT1 (Number of SNPs), Accuracy” column provides the number of SNPs in the first decision tree, and its estimated 10-fold cross-validation accuracy. The “Minimal Number of DTs (Number of SNPs), Accuracy” column gives the minimal number of disjoint decision trees required to achieve the highest accuracy, and the number of SNPs involved, in these trees. The “Number of Robust DTs (Number of SNPs)” column gives the number of decision trees required to achieve robustness and the number of SNPs involved.

## Conclusions

This paper presents a new algorithm called ETHNOPRED that can learn classifiers (each an ensemble of disjoint decision trees) that can identify continental and sub-continental ancestry of a person. While this task is motivated by the challenge of addressing population stratification, it might be useful in-and-of itself, to help determine a person’s ancestry. Applying this approach to downstream association tests/analysis may reduce the false positive and false negative findings by (i) removing the confounding subjects or alternatively, (ii) treating population classification probabilities as a covariate. Our results show that our machine learning approach is able to find distinctions between populations when there is a distinction. Unlike AIMS, our method can accurately distinguish genetically close populations such as Europeans, East Asians, Africans, North Americans, and Kenyans. Unlike many structured association methods, ETHNOPRED is fast and easily extendible to large scale GWASs. Furthermore, ETHNOPRED uses decision trees, which are much simpler and easier to understand than models based on principal component analysis, such as EIGENSTRAT. Note also that decision trees can be easily translated into a set of comprehensible rules, which renders the model completely transparent to the user. While EIGENSTRAT typically uses data from genome wide scans, often involving hundreds of thousands of SNPs, ETHNOPRED uses a small number of SNPs to accurately determine the ancestry of subjects. This means our method is especially useful even in the absence of whole genome (high density) SNP data (*e.g.*, during Stage 2 or Stage 3 of a GWAS). Moreover, as it requires genotypes of only a small number of SNPs, it gets less affected by the genotyping errors compared with methods such as EIGENSTRAT as there is typically a smaller percentage of genotyping errors when dealing with such small number of probes. ETHNOPRED’s ensemble structure makes it robust to missing values, as its multiple trees include enough redundancies that it can return accurate predictions even if it discards some of decision tree while dealing with missing SNPs. We believe that this property of ETHNOPRED makes it beneficial over commonly used methods that use imputation methods for missing values, as those techniques may introduce bias or imperfect estimations. These points all argue that future GWAS studies should consider using ETHNOPRED to estimate the ethnicity of their subjects, towards addressing possible population stratification. While our ETHNOPRED system is focused on predicting ethnicity, it is within the general machine learning framework, of using training information from a group of subjects to produce a personalized classifier, that can provide useful information about subsequent subjects. This paper shows that this framework can work effectively to solve important problems.

## Competing interests

The authors declare that they have no competing interests.

## Authors’ contributions

MH designed the ETHNOPRED method, conducted the experiments and drafted the manuscript; YS prepared the breast cancer dataset, performed genomic control analyses, and offered manuscript edits; JRM offered interface to clinical oncology; PR provided control samples and lineage information for breast cancer study control samples; RG participated in the experimental design and provided manuscript edits; SD conceived the plan to devise ETHNOPRED, offered the breast cancer study data, and offered manuscript edits. All authors read and approved the final manuscript.

## Supplementary Material

Additional file 1 **Appendix A.** 10-fold cross validation accuracy of individual decision trees and ensemble of disjoint decision trees of variable size on continental and sub-continental classification problems; in this Excel© file, you can find the relevant accuracies for each problem on a separate sheet. In each sheet the first column specifies the decision tree index, the second column specifies, the accuracy of the individual decision trees, and the third column specifies the accuracy of the ensemble of disjoint decision trees. Click here for file

Additional file 2 **Appendix B.** ETHNOPRED generated classifier statistics considering accuracy metric and robustness to missing values metrics in different continental and sub-continental population classification problems; this Excel^©^ file presents statistical information of each classification problem in a separate row. Click here for file

Additional file 3 **Appendix C.** Rule-based format of the continental ancestry identification model. Click here for file

Additional file 4 **Appendix D.** Summary statistics of SNPs used by ETHNOPRED method to tackle different continental and sub-continental population classification problems under *accuracy satisfaction condition*; in this excel file, you can find the relevant summary statistics on SNPs used by our method for each problem on a separate sheet. Click here for file

Additional file 5 **Appendix E.** Summary statistics of SNPs used by ETHNOPRED method to tackle different continental and sub-continental population classification problems under *robustness to missing values satisfaction condition*; in this excel file, you can find the relevant summary statistics on SNPs used by our method for each problem on a separate sheet. Click here for file

Additional file 6 **Appendix F.** ETHNOPRED’s output file for a dataset of 696 subjects selected from a breast cancer susceptibility study in Caucasian women of Alberta, Canada [45]. Click here for file

Additional file 7 **Appendix G.** Comparison of self-declared lineage information, EIGENSTRAT’s result and ETHNOPRED’s result on 348 controls selected for a breast cancer susceptibility study in Caucasian women of Alberta, Canada [45]. Click here for file
