# Supplementary material for: ETHNOPRED: a novel machine learning method for accurate continental and sub-continental ancestry identification and population stratification correction
Source: BMC Bioinformatics. 2013 Feb 22;14:61. doi: 10.1186/1471-2105-14-61 (PMC3618021; doi:10.1186/1471-2105-14-61)
Supplement: Additional file 3 — Appendix C. Rule-based format of the continental ancestry identification model. [file 1471-2105-14-61-S3.docx]

**Appendix C: 29 Disjoint Decision Trees/Rule-Bases in Continental Population Identification**

**Decision Tree/Rule-Base 1**

1. IF rs6437783 ϵ {’A_A’} AND rs4835141 ϵ {’A_A’} THEN ethnicity is ‘YRI’
2. IF rs6437783 ϵ {’A_A’} AND rs4835141 ϵ {’A_B’,’B_B’} THEN ethnicity is ‘JPT/CHB’
3. IF rs6437783 ϵ {’A_B’,’B_B’} AND rs735480 ϵ {’A_A’} THEN ethnicity is ‘YRI’
4. IF rs6437783 ϵ {’A_B’,’B_B’} AND rs735480 ϵ {’A_B’,’B_B’} THEN ethnicity is ‘CEU’

**Decision Tree/Rule-Base 2**

1. IF rs35389 ϵ {‘A_A’,’A_B’} AND rs4787645 ϵ {’A_A’} AND rs4847428 ϵ {’A_A’} THEN ethnicity is ‘CEU’
2. IF rs35389 ϵ {‘A_A’,’A_B’} AND rs4787645 ϵ {’A_A’} AND rs4847428 ϵ {’A_B’,’B_B’} THEN ethnicity is ‘YRI’
3. IF rs35389 ϵ {‘A_A’,’A_B’} AND rs4787645 ϵ {’A_B’,’B_B’} THEN ethnicity is ‘JPT/CHB’
4. IF rs35389 ϵ {’B_B’} AND rs1726254 ϵ {‘A_A’, ‘A_B’} THEN ethnicity is ‘CEU’
5. IF rs35389 ϵ {’B_B’} AND rs1726254 ϵ {‘B_B’} THEN ethnicity is ‘YRI’

**Decision Tree/Rule-Base 3**

1. IF rs1986420 ϵ {‘A_A’,’A_B’} AND rs12135904 ϵ {‘A_A’,’A_B’} AND rs1679012 ϵ {‘A_A’} THEN ethnicity is ‘YRI’
2. IF rs1986420 ϵ {‘A_A’,’A_B’} AND rs12135904 ϵ {‘A_A’,’A_B’} AND rs1679012 ϵ {‘A_B’,’B_B’} THEN ethnicity is ‘CEU’
3. IF rs1986420 ϵ {‘A_A’,’A_B’} AND rs12135904 ϵ {‘B_B’} THEN ethnicity is ‘JPT/CHB’
4. IF rs1986420 ϵ {‘B_B’} THEN ethnicity is ‘CEU’

**Decision Tree/Rule-Base 4**

1. IF rs2675345 ϵ {‘A_A’} AND rs9931378 ϵ {‘A_A’,’A_B’} THEN ethnicity is ‘CEU’
2. IF rs2675345 ϵ {‘A_A’} AND rs9931378 ϵ {’B_B’} THEN ethnicity is ‘JPT/CHB’
3. IF rs2675345 ϵ {’A_B’,’B_B’} AND rs6546753 ϵ {‘A_A’,’A_B’} THEN ethnicity is ‘JPT/CHB’
4. IF rs2675345 ϵ {’A_B’,’B_B’} AND rs6546753 ϵ {’B_B’} THEN ethnicity is ‘YRI’

**Decision Tree/Rule-Base 5**

1. IF rs2700392 ϵ {‘A_A’} AND rs1924381 ϵ {‘A_A’} THEN ethnicity is ‘YRI’
2. IF rs2700392 ϵ {‘A_A’} AND rs1924381 ϵ {‘A_B’,’B_B’} THEN ethnicity is ‘CEU’
3. IF rs2700392 ϵ {‘A_B’,’B_B’} AND rs747094 ϵ {‘A_A’,’A_B’} THEN ethnicity is ‘JPT/CHB’
4. IF rs2700392 ϵ {‘A_B’,’B_B’} AND rs747094 ϵ {‘B_B’} AND rs17671597 ϵ {‘A_A’} THEN ethnicity is ‘YRI’
5. IF rs2700392 ϵ {‘A_B’,’B_B’} AND rs747094 ϵ {‘B_B’} AND rs17671597 ϵ {‘A_B’,’B_B’} THEN ethnicity is ‘CEU’

**Decision Tree/Rule-Base 6**

1. IF rs260700 ϵ {‘A_A’,’A_B’} AND rs6451268 ϵ {‘A_A’,’A_B’} AND rs987435 ϵ {‘A_A’} THEN ethnicity is ‘JPT/CHB’
2. IF rs260700 ϵ {‘A_A’,’A_B’} AND rs6451268 ϵ {‘A_A’,’A_B’} AND rs987435 ϵ {‘A_B’,’B_B’} AND rs4242682 ϵ {‘A_A’,’A_B’} THEN ethnicity is ‘YRI’
3. IF rs260700 ϵ {‘A_A’,’A_B’} AND rs6451268 ϵ {‘A_A’,’A_B’} AND rs987435 ϵ {‘A_B’,’B_B’} AND rs4242682 ϵ {’B_B’} THEN ethnicity is ‘CEU’
4. IF rs260700 ϵ {‘A_A’,’A_B’} AND rs6451268 ϵ {’B_B’} AND rs16953500 ϵ {‘A_A’,’A_B’} THEN ethnicity is ‘CEU’
5. IF rs260700 ϵ {‘A_A’,’A_B’} AND rs6451268 ϵ {’B_B’} AND rs16953500 ϵ {‘B_B’} THEN ethnicity is ‘YRI’
6. IF rs260700 ϵ {‘B_B’} AND rs12204275 ϵ {‘A_A’} THEN ethnicity is ‘YRI’
7. IF rs260700 ϵ {‘B_B’} AND rs12204275 ϵ {‘A_B’,’B_B’} THEN ethnicity is ‘JPT/CHB’

**Decision Tree/Rule-Base 7**

1. IF rs260699 ϵ {‘A_A’} AND rs11130791 ϵ {‘A_A’,’A_B’} THEN ethnicity is ‘YRI’
2. IF rs260699 ϵ {‘A_A’} AND rs11130791 ϵ {‘B_B’} THEN ethnicity is ‘JPT/CHB’
3. IF rs260699 ϵ {‘A_B’,’B_B’} AND rs6897135 ϵ {‘A_A’} THEN ethnicity is ‘CEU’
4. IF rs260699 ϵ {‘A_B’,’B_B’} AND rs6897135 ϵ {‘A_B’,’B_B’} AND rs1588040 ϵ {‘A_A’} THEN ethnicity is ‘JPT/CHB’
5. IF rs260699 ϵ {‘A_B’,’B_B’} AND rs6897135 ϵ {‘A_B’,’B_B’} AND rs1588040 ϵ {‘A_B’,’B_B’} AND rs7974633 ϵ {‘A_A’} THEN ethnicity is ‘CEU’
6. IF rs260699 ϵ {‘A_B’,’B_B’} AND rs6897135 ϵ {‘A_B’,’B_B’} AND rs1588040 ϵ {‘A_B’,’B_B’} AND rs7974633 ϵ {‘A_B’,’B_B’} THEN ethnicity is ‘YRI’

**Decision Tree/Rule-Base 8**

1. IF rs260705 ϵ {‘A_A’} AND rs11649653 ϵ {‘A_A’} THEN ethnicity is ‘YRI’
2. IF rs260705 ϵ {‘A_A’} AND rs11649653 ϵ {‘A_B’,’B_B’} THEN ethnicity is ‘JPT/CHB’
3. IF rs260705 ϵ {‘A_B’,’B_B’} AND rs2433354 ϵ {‘A_A’,’A_B’} AND rs2204738 ϵ {‘A_A’,’A_B’} THEN ethnicity is ‘YRI’
4. IF rs260705 ϵ {‘A_B’,’B_B’} AND rs2433354 ϵ {‘A_A’,’A_B’} AND rs2204738 ϵ {‘B_B’} THEN ethnicity is ‘JPT/CHB’
5. IF rs260705 ϵ {‘A_B’,’B_B’} AND rs2433354 ϵ {‘B_B’} AND rs4791868 ϵ {‘A_A’} THEN ethnicity is ‘YRI’
6. IF rs260705 ϵ {‘A_B’,’B_B’} AND rs2433354 ϵ {‘B_B’} AND rs4791868 ϵ {‘A_B’,’B_B’} THEN ethnicity is ‘CEU’

**Decision Tree/Rule-Base 9**

1. IF rs282162 ϵ {‘A_A’,’A_B’} AND rs4825 ϵ {‘A_A’,’A_B’} AND rs1814538 ϵ {‘A_A’} THEN ethnicity is ‘YRI’
2. IF rs282162 ϵ {‘A_A’,’A_B’} AND rs4825 ϵ {‘A_A’,’A_B’} AND rs1814538 ϵ {‘A_B’,’B_B’} THEN ethnicity is ‘CEU’
3. IF rs282162 ϵ {‘A_A’,’A_B’} AND rs4825 ϵ {‘B_B’} AND rs10187056 ϵ {‘A_A’} THEN ethnicity is ‘JPT/CHB’
4. IF rs282162 ϵ {‘A_A’,’A_B’} AND rs4825 ϵ {‘B_B’} AND rs10187056 ϵ {‘A_B’,’B_B’} THEN ethnicity is ‘YRI’
5. IF rs282162 ϵ {‘B_B’} AND rs864386 ϵ {‘A_A’} THEN ethnicity is ‘JPT/CHB’
6. IF rs282162 ϵ {‘B_B’} AND rs864386 ϵ {‘A_B’,’B_B’} THEN ethnicity is ‘CEU’

**Decision Tree/Rule-Base 10**

1. IF rs2002739 ϵ {‘A_A’,’A_B’} AND rs12120383 ϵ {‘A_A’} AND rs7111521 ϵ {‘A_A’} THEN ethnicity is ‘YRI’
2. IF rs2002739 ϵ {‘A_A’,’A_B’} AND rs12120383 ϵ {‘A_A’} AND rs7111521 ϵ {‘A_B’,’B_B’} THEN ethnicity is ‘CEU’
3. IF rs2002739 ϵ {‘A_A’,’A_B’} AND rs12120383 ϵ {‘A_B’,’B_B’} AND rs12921822 ϵ {‘A_A’} THEN ethnicity is ‘JPT/CHB’
4. IF rs2002739 ϵ {‘A_A’,’A_B’} AND rs12120383 ϵ {‘A_B’,’B_B’} AND rs12921822 ϵ {‘A_B’,’B_B’} THEN ethnicity is ‘YRI’
5. IF rs2002739 ϵ {‘B_B’} AND rs2000743 ϵ {‘A_A’,’A_B’} THEN ethnicity is ‘JPT/CHB’
6. IF rs2002739 ϵ {‘B_B’} AND rs2000743 ϵ {’B_B’} THEN ethnicity is ‘CEU’

**Decision Tree/Rule-Base 11**

1. IF rs4722760 ϵ {‘A_A’} AND rs13086858 ϵ {‘A_A’,’A_B’} AND rs7676617 ϵ {‘A_A’} THEN ethnicity is ‘CEU’
2. IF rs4722760 ϵ {‘A_A’} AND rs13086858 ϵ {‘A_A’,’A_B’} AND rs7676617 ϵ {‘A_B’,’B_B’} THEN ethnicity is ‘YRI’
3. IF rs4722760 ϵ {‘A_A’} AND rs13086858 ϵ {‘B_B’} THEN ethnicity is ‘JPT/CHB’
4. IF rs4722760 ϵ {‘A_B’,’B_B’} AND rs1320385 ϵ {‘A_A’,’A_B’} AND rs7215008 ϵ {‘A_A’} THEN ethnicity is ‘JPT/CHB’
5. IF rs4722760 ϵ {‘A_B’,’B_B’} AND rs1320385 ϵ {‘A_A’,’A_B’} AND rs7215008 ϵ {‘A_B’,’B_B’} THEN ethnicity is ‘YRI’
6. IF rs4722760 ϵ {‘A_B’,’B_B’} AND rs1320385 ϵ {’B_B’} AND rs6698919 ϵ {‘A_A’,’A_B’} THEN ethnicity is ‘CEU’
7. IF rs4722760 ϵ {‘A_B’,’B_B’} AND rs1320385 ϵ {’B_B’} AND rs6698919 ϵ {’B_B’} THEN ethnicity is ‘YRI’

**Decision Tree/Rule-Base 12**

1. IF rs2907599 ϵ {‘A_A’} AND rs12036675 ϵ {‘A_A’,’A_B’} THEN ethnicity is ‘YRI’
2. IF rs2907599 ϵ {‘A_A’} AND rs12036675 ϵ {’B_B’} THEN ethnicity is ‘CEU’
3. IF rs2907599 ϵ {‘A_B’,’B_B’} AND rs10814993 ϵ {‘A_A’} AND rs2463383 ϵ {‘A_A’} THEN ethnicity is ‘YRI’
4. IF rs2907599 ϵ {‘A_B’,’B_B’} AND rs10814993 ϵ {‘A_A’} AND rs2463383 ϵ {‘A_B’,’B_B’} THEN ethnicity is ‘JPT/CHB’
5. IF rs2907599 ϵ {‘A_B’,’B_B’} AND rs10814993 ϵ {‘A_B’,’B_B’} AND rs1484213 ϵ {‘A_A’,’A_B’} THEN ethnicity is ‘YRI’
6. IF rs2907599 ϵ {‘A_B’,’B_B’} AND rs10814993 ϵ {‘A_B’,’B_B’} AND rs1484213 ϵ {’B_B’} THEN ethnicity is ‘CEU’

**Decision Tree/Rule-Base 13**

1. IF rs2893312 ϵ {‘A_A’} AND rs868622 ϵ {‘A_A’} THEN ethnicity is ‘JPT/CHB’
2. IF rs2893312 ϵ {‘A_A’} AND rs868622 ϵ {‘A_B’,’B_B’} AND rs2578669 ϵ {‘A_A’,’A_B’} THEN ethnicity is ‘YRI’
3. IF rs2893312 ϵ {‘A_A’} AND rs868622 ϵ {‘A_B’,’B_B’} AND rs2578669 ϵ {’B_B’} THEN ethnicity is ‘CEU’
4. IF rs2893312 ϵ {‘A_B’,’B_B’} AND rs12062528 ϵ {‘A_A’,’A_B’} AND rs519381 ϵ {‘A_A’} THEN ethnicity is ‘JPT/CHB’
5. IF rs2893312 ϵ {‘A_B’,’B_B’} AND rs12062528 ϵ {‘A_A’,’A_B’} AND rs519381 ϵ {‘A_B’,’B_B’} THEN ethnicity is ‘YRI’
6. IF rs2893312 ϵ {‘A_B’,’B_B’} AND rs12062528 ϵ {‘B_B’} AND rs9595066 ϵ {‘A_A’,’A_B’} THEN ethnicity is ‘CEU’
7. IF rs2893312 ϵ {‘A_B’,’B_B’} AND rs12062528 ϵ {‘B_B’} AND rs9595066 ϵ {’B_B’} THEN ethnicity is ‘YRI’

**Decision Tree/Rule-Base 14**

1. IF rs590616 ϵ {‘A_A’} AND rs2605419 ϵ {‘A_A’,’A_B’} THEN ethnicity is ‘JPT/CHB’
2. IF rs590616 ϵ {‘A_A’} AND rs2605419 ϵ {‘B_B’} THEN ethnicity is ‘CEU’
3. IF rs590616 ϵ {‘A_B’,’B_B’} AND rs12895262 ϵ {‘A_A’} THEN ethnicity is ‘CEU’
4. IF rs590616 ϵ {‘A_B’,’B_B’} AND rs12895262 ϵ {‘A_B’,’B_B’} AND rs1909338 ϵ {‘A_A’,’A_B’} AND rs10758940 ϵ {‘A_A’} THEN ethnicity is ‘CEU’
5. IF rs590616 ϵ {‘A_B’,’B_B’} AND rs12895262 ϵ {‘A_B’,’B_B’} AND rs1909338 ϵ {‘A_A’,’A_B’} AND rs10758940 ϵ {‘A_B’,’B_B’} THEN ethnicity is ‘YRI’
6. IF rs590616 ϵ {‘A_B’,’B_B’} AND rs12895262 ϵ {‘A_B’,’B_B’} AND rs1909338 ϵ {’B_B’} THEN ethnicity is ‘JPT/CHB’

**Decision Tree/Rule-Base 15**

1. IF rs533571 ϵ {‘A_A’,’A_B’} AND rs6468344 ϵ {‘A_A’,’A_B’} AND rs11098948 ϵ {‘A_A’} THEN ethnicity is ‘JPT/CHB’
2. IF rs533571 ϵ {‘A_A’,’A_B’} AND rs6468344 ϵ {‘A_A’,’A_B’} AND rs11098948 ϵ {‘A_B’,’B_B’} THEN ethnicity is ‘YRI’
3. IF rs533571 ϵ {‘A_A’,’A_B’} AND rs6468344 ϵ {‘B_B’} AND rs2029623 ϵ {‘A_A’} THEN ethnicity is ‘YRI’
4. IF rs533571 ϵ {‘A_A’,’A_B’} AND rs6468344 ϵ {‘B_B’} AND rs2029623 ϵ {‘A_B’,’B_B’} THEN ethnicity is ‘CEU’
5. IF rs533571 ϵ {‘B_B’} AND rs4577845 ϵ {‘A_A’,’A_B’} THEN ethnicity is ‘JPT/CHB’
6. IF rs533571 ϵ {‘B_B’} AND rs4577845 ϵ {‘B_B’} THEN ethnicity is ‘CEU’

**Decision Tree/Rule-Base 16**

1. IF rs260709 ϵ {‘A_A’,’A_B’} AND rs11691947 ϵ {‘A_A’,’A_B’} AND rs7158108 ϵ {‘A_A’,’A_B’} THEN ethnicity is ‘CEU’
2. IF rs260709 ϵ {‘A_A’,’A_B’} AND rs11691947 ϵ {‘A_A’,’A_B’} AND rs7158108 ϵ {‘B_B’} THEN ethnicity is ‘YRI’
3. IF rs260709 ϵ {‘A_A’,’A_B’} AND rs11691947 ϵ {‘B_B’} AND rs4705115 ϵ {‘A_A’} THEN ethnicity is ‘JPT/CHB’
4. IF rs260709 ϵ {‘A_A’,’A_B’} AND rs11691947 ϵ {‘B_B’} AND rs4705115 ϵ {‘A_B’,’B_B’} AND rs2214965 ϵ {‘A_A’} THEN ethnicity is ‘CEU’
5. IF rs260709 ϵ {‘A_A’,’A_B’} AND rs11691947 ϵ {‘B_B’} AND rs4705115 ϵ {‘A_B’,’B_B’} AND rs2214965 ϵ {‘A_B’,’B_B’} THEN ethnicity is ‘YRI’
6. IF rs260709 ϵ {’B_B’} AND rs1437724 ϵ {‘A_A’} THEN ethnicity is ‘YRI’
7. IF rs260709 ϵ {’B_B’} AND rs1437724 ϵ {‘A_B’,’B_B’} THEN ethnicity is ‘JPT/CHB’

**Decision Tree/Rule-Base 17**

1. IF rs6534999 ϵ {‘A_A’,’A_B’} AND rs7689609 ϵ {‘A_A’,’A_B’} THEN ethnicity is ‘CEU’
2. IF rs6534999 ϵ {‘A_A’,’A_B’} AND rs7689609 ϵ {’B_B’} AND rs1450428 ϵ {‘A_A’} THEN ethnicity is ‘JPT/CHB’
3. IF rs6534999 ϵ {‘A_A’,’A_B’} AND rs7689609 ϵ {’B_B’} AND rs1450428 ϵ {‘A_B’,’B_B’} THEN ethnicity is ‘YRI’
4. IF rs6534999 ϵ {’B_B’} AND rs12542990 ϵ {‘A_A’,’A_B’} AND rs7551132 ϵ {‘A_A’} THEN ethnicity is ‘CEU’
5. IF rs6534999 ϵ {’B_B’} AND rs12542990 ϵ {‘A_A’,’A_B’} AND rs7551132 ϵ {‘A_B’,’B_B’} THEN ethnicity is ‘YRI’
6. IF rs6534999 ϵ {’B_B’} AND rs12542990 ϵ {’B_B’} THEN ethnicity is ‘JPT/CHB’

**Decision Tree/Rule-Base 18**

1. IF rs4832501 ϵ {‘A_A’} AND rs2200402 ϵ {‘A_A’,’A_B’} AND rs2546001 ϵ {‘A_A’} THEN ethnicity is ‘YRI’
2. IF rs4832501 ϵ {‘A_A’} AND rs2200402 ϵ {‘A_A’,’A_B’} AND rs2546001 ϵ {‘A_B’,’B_B’} THEN ethnicity is ‘CEU’
3. IF rs4832501 ϵ {‘A_A’} AND rs2200402 ϵ {’B_B’} AND rs8091955 ϵ {‘A_A’,’A_B’} THEN ethnicity is ‘JPT/CHB’
4. IF rs4832501 ϵ {‘A_A’} AND rs2200402 ϵ {’B_B’} AND rs8091955 ϵ {’B_B’} THEN ethnicity is ‘YRI’
5. IF rs4832501 ϵ {‘A_B’,’B_B’} AND rs931410 ϵ {‘A_A’} AND rs1099968 ϵ {‘A_A’,’A_B’} AND rs7921545 ϵ {‘A_A’,’A_B’} THEN ethnicity is ‘YRI’
6. IF rs4832501 ϵ {‘A_B’,’B_B’} AND rs931410 ϵ {‘A_A’} AND rs1099968 ϵ {‘A_A’,’A_B’} AND rs7921545 ϵ {’B_B’} THEN ethnicity is ‘JPT/CHB’
7. IF rs4832501 ϵ {‘A_B’,’B_B’} AND rs931410 ϵ {‘A_A’} AND rs1099968 ϵ {‘B_B’} THEN ethnicity is ‘CEU’
8. IF rs4832501 ϵ {‘A_B’,’B_B’} AND rs931410 ϵ {‘A_B’,’B_B’} THEN ethnicity is ‘CEU’

**Decision Tree/Rule-Base 19**

1. IF rs1437787 ϵ {‘A_A’} AND rs11170991 ϵ {‘A_A’,’A_B’} THEN ethnicity is ‘JPT/CHB’
2. IF rs1437787 ϵ {‘A_A’} AND rs11170991 ϵ {‘B_B’} THEN ethnicity is ‘CEU’
3. IF rs1437787 ϵ {‘A_B’,’B_B’} AND rs1805972 ϵ {‘A_A’} AND rs2422098 ϵ {‘A_A’} THEN ethnicity is ‘YRI’
4. IF rs1437787 ϵ {‘A_B’,’B_B’} AND rs1805972 ϵ {‘A_A’} AND rs2422098 ϵ {‘A_B’,’B_B’} THEN ethnicity is ‘CEU’
5. IF rs1437787 ϵ {‘A_B’,’B_B’} AND rs1805972 ϵ {‘A_B’,’B_B’} AND rs9821525 ϵ {‘A_A’} THEN ethnicity is ‘JPT/CHB’
6. IF rs1437787 ϵ {‘A_B’,’B_B’} AND rs1805972 ϵ {‘A_B’,’B_B’} AND rs9821525 ϵ {‘A_B’,’B_B’} AND rs12511245 ϵ {‘A_A’,’A_B’} THEN ethnicity is ‘YRI’
7. IF rs1437787 ϵ {‘A_B’,’B_B’} AND rs1805972 ϵ {‘A_B’,’B_B’} AND rs9821525 ϵ {‘A_B’,’B_B’} AND rs12511245 ϵ {‘B_B’} THEN ethnicity is ‘CEU’

**Decision Tree/Rule-Base 20**

1. IF rs4749305 ϵ {‘A_A’} AND rs2631899 ϵ {‘A_A’,’A_B’} THEN ethnicity is ‘JPT/CHB’
2. IF rs4749305 ϵ {‘A_A’} AND rs2631899 ϵ {‘B_B’} THEN ethnicity is ‘YRI’
3. IF rs4749305 ϵ {‘A_B’,’B_B’} AND rs7956796 ϵ {‘A_A’,’A_B’} THEN ethnicity is ‘CEU’
4. IF rs4749305 ϵ {‘A_B’,’B_B’} AND rs7956796 ϵ {‘B_B’} AND rs4877791ϵ {‘A_A’,’A_B’} AND rs1932366 ϵ {‘A_A’} THEN ethnicity is ‘CEU’
5. IF rs4749305 ϵ {‘A_B’,’B_B’} AND rs7956796 ϵ {‘B_B’} AND rs4877791 ϵ {‘A_A’,’A_B’} AND rs1932366 ϵ {‘A_B’,’B_B’} THEN ethnicity is ‘YRI’
6. IF rs4749305 ϵ {‘A_B’,’B_B’} AND rs7956796 ϵ {‘B_B’} AND rs4877791 ϵ {‘B_B’} THEN ethnicity is ‘JPT/CHB’

**Decision Tree/Rule-Base 21**

1. IF rs542405 ϵ {‘A_A’,’A_B’} AND rs6977118 ϵ {‘A_A’} AND rs11563620 ϵ {‘A_A’} THEN ethnicity is ‘YRI’
2. IF rs542405 ϵ {‘A_A’,’A_B’} AND rs6977118 ϵ {‘A_A’} AND rs11563620 ϵ {‘A_B’,’B_B’} THEN ethnicity is ‘CEU’
3. IF rs542405 ϵ {‘A_A’,’A_B’} AND rs6977118 ϵ {‘A_B’,’B_B’} THEN ethnicity is ‘JPT/CHB’
4. IF rs542405 ϵ {‘B_B’} AND rs2305858 ϵ {‘A_A’,’A_B’} AND rs2040704 ϵ {‘A_A’} THEN ethnicity is ‘JPT/CHB’
5. IF rs542405 ϵ {‘B_B’} AND rs2305858 ϵ {‘A_A’,’A_B’} AND rs2040704 ϵ {‘A_B’,’B_B’} THEN ethnicity is ‘YRI’
6. IF rs542405 ϵ {‘B_B’} AND rs2305858 ϵ {‘B_B’} THEN ethnicity is ‘CEU’

**Decision Tree/Rule-Base 22**

1. IF rs738276 ϵ {‘A_A’} AND rs4615248 ϵ {‘A_A’,’A_B’} AND rs12681671 ϵ {‘A_A’} THEN ethnicity is ‘YRI’
2. IF rs738276 ϵ {‘A_A’} AND rs4615248 ϵ {‘A_A’,’A_B’} AND rs12681671 ϵ {‘A_B’,’B_B’} THEN ethnicity is ‘CEU’
3. IF rs738276 ϵ {‘A_A’} AND rs4615248 ϵ {‘B_B’} THEN ethnicity is ‘JPT/CHB’
4. IF rs738276 ϵ {‘A_B’,’B_B’} AND rs7983489 ϵ {‘A_A’} AND rs6035761 ϵ {‘A_A’} THEN ethnicity is ‘YRI’
5. IF rs738276 ϵ {‘A_B’,’B_B’} AND rs7983489 ϵ {‘A_A’} AND rs6035761 ϵ {‘A_B’,’B_B’} THEN ethnicity is ‘CEU’
6. IF rs738276 ϵ {‘A_B’,’B_B’} AND rs7983489 ϵ {‘A_B’,’B_B’} AND rs2324520 ϵ {‘A_A’,’A_B’} AND rs3181372 ϵ {‘A_A’,’A_B’} THEN ethnicity is ‘YRI’
7. IF rs738276 ϵ {‘A_B’,’B_B’} AND rs7983489 ϵ {‘A_B’,’B_B’} AND rs2324520 ϵ {‘A_A’,’A_B’} AND rs3181372 ϵ {‘B_B’} THEN ethnicity is ‘CEU’
8. IF rs738276 ϵ {‘A_B’,’B_B’} AND rs7983489 ϵ {‘A_B’,’B_B’} AND rs2324520 ϵ {‘B_B’} THEN ethnicity is ‘JPT/CHB’

**Decision Tree/Rule-Base 23**

1. IF rs943773 ϵ {‘A_A’} AND rs4789182 ϵ {‘A_A’} THEN ethnicity is ‘YRI’
2. IF rs943773 ϵ {‘A_A’} AND rs4789182 ϵ {‘A_B’,’B_B’} THEN ethnicity is ‘JPT/CHB’
3. IF rs943773 ϵ {‘A_B’,’B_B’} AND rs702032 ϵ {‘A_A’,’A_B’} AND rs6926482 ϵ {‘A_A’} THEN ethnicity is ‘JPT/CHB’
4. IF rs943773 ϵ {‘A_B’,’B_B’} AND rs702032 ϵ {‘A_A’,’A_B’} AND rs6926482 ϵ {‘A_B’,’B_B’} AND rs2490385 ϵ {‘A_A’} THEN ethnicity is ‘CEU’
5. IF rs943773 ϵ {‘A_B’,’B_B’} AND rs702032 ϵ {‘A_A’,’A_B’} AND rs6926482 ϵ {‘A_B’,’B_B’} AND rs2490385 ϵ {‘A_B’,’B_B’} THEN ethnicity is ‘YRI’
6. IF rs943773 ϵ {‘A_B’,’B_B’} AND rs702032 ϵ {‘B_B’} THEN ethnicity is ‘CEU’

**Decision Tree/Rule-Base 24**

1. IF rs8097206 ϵ {‘A_A’,’A_B’} AND rs39639 ϵ {‘A_A’} AND rs9866028 ϵ {‘A_A’} THEN ethnicity is ‘YRI’
2. IF rs8097206 ϵ {‘A_A’,’A_B’} AND rs39639 ϵ {‘A_A’} AND rs9866028 ϵ {‘A_B’,’B_B’} THEN ethnicity is ‘CEU’
3. IF rs8097206 ϵ {‘A_A’,’A_B’} AND rs39639 ϵ {‘A_B’,’B_B’} AND rs4713659 ϵ {‘A_A’,’A_B’} AND rs271626 ϵ {‘A_A’,’A_B’} THEN ethnicity is ‘YRI’
4. IF rs8097206 ϵ {‘A_A’,’A_B’} AND rs39639 ϵ {‘A_B’,’B_B’} AND rs4713659 ϵ {‘A_A’,’A_B’} AND rs271626 ϵ {‘B_B’} THEN ethnicity is ‘CEU’
5. IF rs8097206 ϵ {‘A_A’,’A_B’} AND rs39639 ϵ {‘A_B’,’B_B’} AND rs4713659 ϵ {‘B_B’} THEN ethnicity is ‘JPT/CHB’
6. IF rs8097206 ϵ {‘B_B’} AND rs12641411 ϵ {‘A_A’,’A_B’} THEN ethnicity is ‘CEU’
7. IF rs8097206 ϵ {‘B_B’} AND rs12641411 ϵ {‘B_B’} THEN ethnicity is ‘JPT/CHB’

**Decision Tree/Rule-Base 25**

1. IF rs10851731 ϵ {‘A_A’} AND rs6560084 ϵ {‘A_A’,’A_B’} THEN ethnicity is ‘CEU’
2. IF rs10851731 ϵ {‘A_A’} AND rs6560084 ϵ {‘B_B’} THEN ethnicity is ‘YRI’
3. IF rs10851731 ϵ {‘A_B’,’B_B’} AND rs11085023 ϵ {‘A_A’,’A_B’} AND rs277639 ϵ {‘A_A’} THEN ethnicity is ‘CEU’
4. IF rs10851731 ϵ {‘A_B’,’B_B’} AND rs11085023 ϵ {‘A_A’,’A_B’} AND rs277639 ϵ {‘A_B’,’B_B’} AND rs7677859 ϵ {‘A_A’} THEN ethnicity is ‘JPT/CHB’
5. IF rs10851731 ϵ {‘A_B’,’B_B’} AND rs11085023 ϵ {‘A_A’,’A_B’} AND rs277639 ϵ {‘A_B’,’B_B’} AND rs7677859 ϵ {‘A_B’,’B_B’} THEN ethnicity is ‘YRI’
6. IF rs10851731 ϵ {‘A_B’,’B_B’} AND rs11085023 ϵ {‘B_B’} THEN ethnicity is ‘JPT/CHB’

**Decision Tree/Rule-Base 26**

1. IF rs2228511 ϵ {‘A_A’,’A_B’} AND rs738987 ϵ {‘A_A’} AND rs560767 ϵ {‘A_A’,’A_B’} THEN ethnicity is ‘JPT/CHB’
2. IF rs2228511 ϵ {‘A_A’,’A_B’} AND rs738987 ϵ {‘A_A’} AND rs560767 ϵ {‘B_B’} THEN ethnicity is ‘YRI’
3. IF rs2228511 ϵ {‘A_A’,’A_B’} AND rs738987 ϵ {‘A_B’,’B_B’} AND rs2967391 ϵ {‘A_A’,’A_B’} THEN ethnicity is ‘YRI’
4. IF rs2228511 ϵ {‘A_A’,’A_B’} AND rs738987 ϵ {‘A_B’,’B_B’} AND rs2967391 ϵ {‘B_B’} THEN ethnicity is ‘CEU’
5. IF rs2228511 ϵ {‘B_B’} AND rs2500090 ϵ {‘A_A’,’A_B’} THEN ethnicity is ‘CEU’
6. IF rs2228511 ϵ {‘B_B’} AND rs2500090 ϵ {‘B_B’} THEN ethnicity is ‘YRI’

**Decision Tree/Rule-Base 27**

1. **IF rs1942885 ϵ {‘A_A’,’A_B’} AND rs11903376 ϵ {‘A_A’} THEN ethnicity is ‘CEU’**
2. **IF rs1942885 ϵ {‘A_A’,’A_B’} AND rs11903376 ϵ {‘A_B’,’B_B’} AND rs2035247 ϵ {‘A_A’} THEN ethnicity is ‘CEU’**
3. **IF rs1942885 ϵ {‘A_A’,’A_B’} AND rs11903376 ϵ {‘A_B’,’B_B’} AND rs2035247 ϵ {‘A_B’,’B_B’} AND rs6679430 ϵ {‘A_A’,’A_B’} THEN ethnicity is ‘YRI’**
4. **IF rs1942885 ϵ {‘A_A’,’A_B’} AND rs11903376 ϵ {‘A_B’,’B_B’} AND rs2035247 ϵ {‘A_B’,’B_B’} AND rs6679430 ϵ {‘B_B’} THEN ethnicity is ‘JPT/CHB’**
5. IF rs1942885 ϵ {‘B_B’} AND rs12677218 ϵ {‘A_A’,’A_B’} AND rs2849372 ϵ {‘A_A’,’A_B’} THEN ethnicity is ‘YRI’
6. IF rs1942885 ϵ {‘B_B’} AND rs12677218 ϵ {‘A_A’,’A_B’} AND rs2849372 ϵ {‘B_B’} THEN ethnicity is ‘CEU’
7. IF rs1942885 ϵ {‘B_B’} AND rs12677218 ϵ {‘B_B’} THEN ethnicity is ‘JPT/CHB’

**Decision Tree/Rule-Base 28**

1. IF rs260711 ϵ {‘A_A’} AND rs10998087 ϵ {‘A_A’,’A_B’} THEN ethnicity is ‘JPT/CHB’
2. IF rs260711 ϵ {‘A_A’} AND rs10998087 ϵ {‘B_B’} THEN ethnicity is ‘YRI’
3. IF rs260711 ϵ {‘A_B’,’B_B’} AND rs1117382 ϵ {‘A_A’,’A_B’} THEN ethnicity is ‘CEU’
4. IF rs260711 ϵ {‘A_B’,’B_B’} AND rs1117382 ϵ {‘B_B’} AND rs7376483 ϵ {‘A_A’,’A_B’} AND rs12446781 ϵ {‘A_A’} THEN ethnicity is ‘CEU’
5. IF rs260711 ϵ {‘A_B’,’B_B’} AND rs1117382 ϵ {‘B_B’} AND rs7376483 ϵ {‘A_A’,’A_B’} AND rs12446781 ϵ {‘A_B’,’B_B’} THEN ethnicity is ‘YRI’
6. IF rs260711 ϵ {‘A_B’,’B_B’} AND rs1117382 ϵ {‘B_B’} AND rs7376483 ϵ {‘B_B’} THEN ethnicity is ‘JPT/CHB’

**Decision Tree/Rule-Base 29**

1. IF rs1250253 ϵ {‘A_A’,’A_B’} AND rs1478446 ϵ {‘A_A’,’A_B’} AND rs2673884 ϵ {‘A_A’,’A_B’} AND rs158512 ϵ {‘A_A’} THEN ethnicity is ‘CEU’
2. IF rs1250253 ϵ {‘A_A’,’A_B’} AND rs1478446 ϵ {‘A_A’,’A_B’} AND rs2673884 ϵ {‘A_A’,’A_B’} AND rs158512 ϵ {‘A_B’,’B_B’} THEN ethnicity is ‘YRI’
3. IF rs1250253 ϵ {‘A_A’,’A_B’} AND rs1478446 ϵ {‘A_A’,’A_B’} AND rs2673884 ϵ {‘B_B’} THEN ethnicity is ‘JPT/CHB’
4. IF rs1250253 ϵ {‘A_A’,’A_B’} AND rs1478446 ϵ {‘B_B’} AND rs2506898 ϵ {‘A_A’,’A_B’} THEN ethnicity is ‘CEU’
5. IF rs1250253 ϵ {‘A_A’,’A_B’} AND rs1478446 ϵ {‘B_B’} AND rs2506898 ϵ {‘B_B’} THEN ethnicity is ‘YRI’
6. IF rs1250253 ϵ {‘B_B’} AND rs6763648 ϵ {‘A_A’} THEN ethnicity is ‘JPT/CHB’
7. IF rs1250253 ϵ {‘B_B’} AND rs6763648 ϵ {‘A_B’,’B_B’} THEN ethnicity is ‘YRI’
